# Sexual-Related Knowledge, School and Family Sexuality Education and Its Association with Experience of Sexual Intercourse among Vocational Secondary School Students in China

**DOI:** 10.3390/children9081206

**Published:** 2022-08-11

**Authors:** Yuhang Fang, Yujia Zheng, Yan Jin, Chunyan Yu, Xiayun Zuo, Qiguo Lian, Chaohua Lou, Lihe Li, Ping Hong, Xiaowen Tu

**Affiliations:** 1 NHC Key Laboratory of Reproduction Regulation, Shanghai Institute for Biomedical and Pharmaceutical Technologies, Shanghai 200237, China; 2 Shaanxi Xin Hang Public Health Research Center, Xi’an 710065, China; 3 China Family Planning Association, Beijing 100035, China

**Keywords:** sexual-related knowledge, sexual behavior, sexuality education, vocational secondary school, adolescents, China

## Abstract

Objectives: To investigate the associations between sexual-related knowledge, access to school and family sexuality education, and the experience of sexual intercourse, in order to make recommendations on sexuality education for vocational secondary school students in China. Methods: A cross-sectional study was conducted among 3180 vocational secondary school students in the Shanghai municipality and the Shaanxi province, China. Data were collected through an online, electronic questionnaire, which included socio-demographics, sexual-related knowledge, sources of information, and sexual-related behaviors. Results: The score on sexual-related knowledge among girls (53.8) is higher than that of boys (48.8), and that of participants from Shanghai (55.2) is higher than their counterparts from Shaanxi (47.6). The proportions of girls (70% and 41.7%, respectively) and participants from Shanghai (65% and 35.7%, respectively) who reported acquiring sexual information from their schools/teachers and parents are higher than that of boys (54.3% and 21.0%, respectively) and their counterparts from Shaanxi (59.7% and 27.4%, respectively). About 6% of participants had experienced sexual intercourse and 10% had watched porn actively. Experience of sexual intercourse was associated with higher sexual-related knowledge scores (OR = 1.01, 95% CI: 1.00–1.02) and active porn watching (OR = 2.63, 95% CI: 1.79–3.84) but was not associated with school and family sexuality education. Conclusions: Vocational secondary school students had poor sexual knowledge and inadequate school and family sexuality education. School and family-based comprehensive sexuality education should be promoted among vocational secondary school students.

## 1. Introduction

Adolescent sexual and reproductive health (ASRH) is a global concern. The World Health Organization (WHO) reports that about 12 million adolescent girls aged 15–19 give birth every year in low- and middle-income countries, half of all sexually transmitted diseases (STDs) in America were reported in adolescents aged 15–24, and approximately 1.8 million adolescents are living with human immunodeficiency virus (HIV) worldwide [1,2]. Pregnancy and childbirth complications are estimated to be the leading cause of death among 15- to 19-year-old girls worldwide [3]. Adolescents aged 15–19 years have greater maternal health risks than women just a few years older [4]. Relative to older women, adolescents are more likely to experience pre-eclampsia, eclampsia, puerperal endometritis, and systemic infections [5], and they have a higher risk of preterm birth, stillbirths, low birth weight, and neonatal mortality [6]. In sub-Saharan Africa, studies carried out on women with obstetric fistulae have reported that 35–40% of them first developed the condition as adolescents [7]. In China, an increasing number of adolescents are engaging in sexual activity without any knowledge of how to better protect themselves [8]. As a result, unintended pregnancy, induced abortion, and STDs, including HIV infection among young people, have become major public health issues. For example, about 22.4% of youths aged 15–24 had the experience of sexual intercourse, with boys higher than girls [9], and developed regions higher than less developed regions [10]. Additionally, 25% of sexually active youth were infected with genital chlamydia within one year of their sexual intercourse, and about 21.3% of sexually active girls experienced an unintended pregnancy, of which 90.9% had abortions [11].

Among Chinese adolescents, vocational secondary school students (VSSS) are a vulnerable sub-group with a higher risk of sexual-related behaviors [12,13]. Based on the scores of the high school enrollment examination, junior high school students with lower scores (approximately 40%) will attend vocational secondary school in China. Compared with their peers in regular senior high schools, VSSS have less academic pressure to enter a higher-education school, have more opportunity to make contact with society, and are more likely to engage in smoking, drinking, and sexual intercourse [12,14]. For example, VSSS were 3 times more likely to engage in smoking, drinking, and fighting than their counterparts in regular senior high school [15]. However, sexuality education among VSSS has been neglected. 

In traditional Chinese society, talking about sex in public or between different generations is a taboo [16]. Confucianism advocated, “Cherish Heaven Laws, Extinguishing Human Desires.” Only sex within marriage for the purpose of reproduction was approved, and sex openness was treated as a threat to society and religion [17]. Under traditional Chinese social norms, abstinence among adolescents is encouraged, and parents generally avoid discussing sex-related issues directly with their children because these issues are considered to be personal, sensitive, and embarrassing [16]. With the rapid development of society and the economy since the opening-up policy in the late 1970s, people’s attitudes towards sexuality and sexual behaviors have changed a lot, especially among young people [18]. However, many parents still believe that it is not necessary to provide sexuality education to their children [19]. For example, findings from a study conducted among senior high school and college students in 11 provinces in China show that about 75.29% of high school students reported that they had never discussed sex-related topics with their parents [20]. The practice of sexuality education is minimized within schools, and western China lags far behind eastern China in the promotion of comprehensive sexuality education [8,21]. This is on account of western China’s more conservative culture, its lower level of economic development, and its relative lack of faculty resources [21]. Most schools integrated sexuality education into subjects such as psychology, biology, and moral education using lectures, videos, in-class discussion, and class meetings as teaching methods [22]. The contents of the current school sexuality education, if any, mainly cover puberty development, HIV/AIDS prevention, self-protection, and heterosexual relationships, but rarely cover topics on gender, sexual behavior, contraception, pregnancy, induced abortion, and sexual rights [22,23]. Fortunately, the Chinese government has attached growing importance to sexuality education for adolescents. The last revised Law on Protection of Minors (2020) clearly states that schools and kindergartens should provide age-appropriate sexuality education for minors. We aim to investigate the status of sexual-related knowledge, access to school and family sexuality education, and its association with sexual intercourse among VSSS in different regions and genders, so as to provide evidence and recommendations for promoting sexuality education among VSSS in China.

## 2. Materials and Methods

### 2.1. Study Design, Participants and Procedure

This is a cross-sectional study conducted in 6 vocational secondary schools in the Shanghai Municipality and Shaanxi Province from March through April 2021. Shanghai is the most developed and largest city in eastern China, while Shaanxi is a typically less developed, inland province in western China. Three vocational secondary schools were selected from the central urban, suburban, and outer suburb areas of Shanghai, and three were selected from three underdeveloped cities in Shaanxi, respectively. In each selected school, students from three or more majors were selected by cluster sampling with equal proportions of gender and grade.

The study protocol was reviewed and approved by the Medical Ethical Committee of Shanghai Institute of Biological and Pharmaceutical Technologies (formerly named Shanghai Institute of Planned Parenthood Research, No. PJ2021-24). Informed consent or assent was obtained from all participants and their legal guardians. 

Data were collected with anonymous, self-administered questionnaires via an online electronic questionnaire platform. Two trained investigators were present, who were responsible for introducing the survey, distributing the questionnaire links, explaining any questions which the participants might have, and distributing gifts after the procedure. 

Among 3253 eligible students, 3237 participated in the study with the response rate of 99.5%. After the exclusion of 57 duplicate records, a total of 3180 participants were included in the final analysis.

### 2.2. Measurement

The questionnaire was developed based on the previous study among adolescents [24,25].

#### 2.2.1. Sexual-Related Knowledge and Sources of Information

Sexual-related knowledge with four dimensions was assessed, namely sexual physiology (5 single-choice/answer questions), STD/AIDS (8 single-choice/answer questions, 1 multiple-choice/answer question), contraception (1 single-choice/answer question, 1 multiple-choice/answer question), and sexual abuse (2 single-choice/answer questions). For each item of question, one point was awarded for correct answers and zero points for incorrect answers or unknown responses. (See Table S1 for specific questions and points.) The sum of the points for each dimension was converted into a score on a scale of 0 to 100, with higher scores indicating higher levels of knowledge.

Sources of sexual-related information including school/teacher, parents, internet, etc., were measured through a multiple-choice/answer question. For school and family sexuality education, the education topics were further collected through multiple-choice/answer questions. In the binary-logistic-regression analysis, we summed up the topics of school or family sexuality education reported by each participant and included the number of topics as a continuous variable.

#### 2.2.2. Sexual-Related Behaviors

The information on heterosexual intercourse (Yes/No) and active porn watching (Yes/No) were collected through single-choice/answer questions. 

#### 2.2.3. Covariates

Covariates assessed in this study include: age, gender, region, local residents or not, living with parents or not, parents’ marital status, family socio-economic status, peers’ romantic relationships (None of them/Few/About half/Most/All/Unknown), and attitudes toward middle school students’ sexual intercourse (“Completely accept”, “Understandable”, “Unsure”, and “Unacceptable”). 

### 2.3. Data Analysis

Stata/SE 15.1 (StataCorp LLC, College Station, TX, USA) was used for data management and analysis. A chi-square test was used to compare the subgroup differences in the distribution of demographic characteristics, information sources, and sexual-related behaviors. A *t*-test was used to compare scores on sexual-related knowledge between groups. Finally, binary-logistic-regression analysis was used to explore the association between sexual-related knowledge, school and family sexuality education, active porn watching, and experience of sexual intercourse, adjusting for covariates.

## 3. Results

### 3.1. Socio-Demographics of Study Participants

As shown in Table 1, among the 3180 participants, Shanghai and Shaanxi participants accounted for 49.7% and 50.3%, respectively, and the average age of the girls (17.0 years) was slightly elder than the boys’ (16.8 years). The majority of participants were local residents (80.6%) and lived with their parents (76.6%). About 21.6% of the participants reported that their parents were divorced/separated/widowed. Most of participants (63.4%) believed that their family economic level was medium. Compared with girls, boys were more likely to be local residents and have low family economic level (84.3% and 12.8% vs. 77.2% and 8.5%, respectively) but were less likely to live with parents (73.5% vs. 80.0%).

### 3.2. Knowledge Score and Sources of Knowledge and Information

The total, mean score about sexual-related knowledge of participants was 51.36. Significant gender and regional differences were observed in the total score and the four-dimensions’ score. Girls scored between 2.3 and 16.3 points higher than boys, while participants in Shanghai scored between 4.1 and 11.0 points higher than their counterparts in Shaanxi for the four dimensions of knowledge (Table 2).

**Figure 1:**
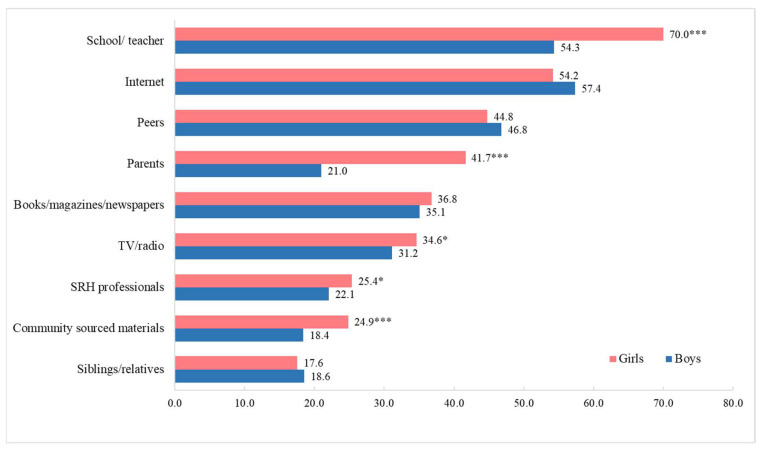
Sources of sexual-related information by gender (%; *: <0.05; ***: <0.001).

**Figure 2:**
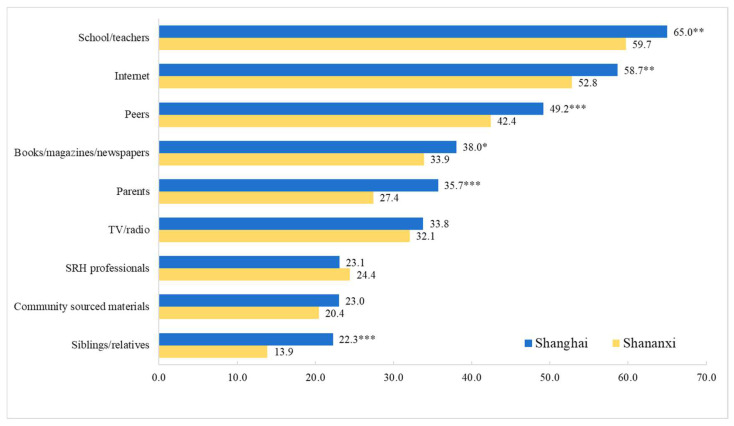
Sources of sexual-related information by region (%; *: <0.05; **: <0.01; ***: <0.001).

School/teacher, Internet, and peers were the top three sources of sexual-related information for participants, and there were gender and regional differences. The proportion of girls who reported obtaining information through a school/teacher was significantly higher than that of boys (70.0% vs. 54.3%). Girls also reported a higher proportion of parents, TV/radio, SRH professionals, and community-sourced materials than boys (Figure 1). A higher proportion of participants from Shanghai reported acquiring sexual-related information from a school/teacher, Internet, peers, and parents, etc., than their counterparts from Shaanxi, except for three sources (TV/radio, SRH professionals, and community-sourced materials). For example, 65% of participants from Shanghai reported acquiring SRH information from a school/teacher, versus 59.7% of their counterparts from Shaanxi (Figure 2).

### 3.3. Topics Covered in Family and School Sexuality Education

The top three topics of family sexuality education were interpersonal relationships, puberty development and health care, and romantic relationships, with higher proportions reported by girls (61.2%, 57.4%, and 46.3%, respectively) than boys (55.2%, 40.2%, and 33.1%, respectively) and a higher proportion of interpersonal relationships but a lower proportion of romantic relationships reported by participants from Shaanxi (63.2% and 35.8%, respectively) than their counterparts from Shanghai (53.3% and 43.9%, respectively). The proportion of other topics covered by family sexuality education ranged from 1.8% to 27.4%. In general, participants from Shaanxi were less likely to access family sexuality education than their counterparts from Shanghai (Table 3).

In terms of the topics covered in school sexuality education, more than 60% of participants reported puberty development/health care, saying no to drugs, interpersonal relationships, values/decision making, and STDs/AIDS prevention, but less than one-third of them reported reproduction and contraception. Obvious regional disparity was observed with higher coverage of topics in Shanghai than in Shaanxi, except for STDs prevention and planning for the future (Table 3).

### 3.4. Sexual-Related Behaviors

Gender and regional disparities were observed in the experience of sexual-related behaviors. Boys and participants from Shanghai were more likely to report heterosexual intercourse (8.9% and 7.0%, respectively) and watching porn actively (15.4% and 14.9%, respectively) than girls (2.8% and 5.1%, respectively) and their counterparts from Shaanxi (4.6% and 5.3%, respectively).

### 3.5. Association of Sexual-Related Knowledge, School and Family Sexuality Education, and Active Porn Watching with Sexual Intercourse 

Using logistic regression analysis to explore factors associated with the experience of sexual intercourse, we found that the risk of sexual intercourse was not associated with the number of topics covered in family and school sexuality education after controlling for covariates. Sexual-related knowledge (OR = 1.01, 95% CI: 1.00–1.02) and active porn watching (OR = 2.63, 95% CI: 1.79–3.84) were positively associated with the risk of sexual intercourse (Table 4). 

## 4. Discussion

The finding of the study shows that vocational secondary school students had poor sexual knowledge and inadequate school and family sexuality education, especially boys and participants from the Shaanxi Province. Experience of sexual intercourse was associated with higher sexual-related knowledge and active porn watching, but was not associated with schools and family sexuality education.

Overall, VSSS had poor sexual-related knowledge, especially knowledge about sexual physiology and contraception. In this study, 62% of the participants reported schools/teachers as a source of sexual-related information, and only 32% of the participants reported parents as a SRH information source. The topics of school and family sexuality education mainly covered puberty development, interpersonal relationships, and STD/AIDS prevention, but other sexual-related topics were covered poorly, which is in line with the findings observed in previous studies [9,26]. Insufficient school and family sexuality education may have contributed to poor knowledge among VSSS (Table S2). 

Consistent with previous studies [27,28], regional and gender differences in sexual-related knowledge were observed in this study, with girls better than boys and the eastern areas better than the western areas. This finding can be explained by the significant gender and regional difference in source of information and topics covered in school and family sexuality education. In this study, girls and participants from the eastern areas were significantly more likely to acquire sexual-related knowledge from their school and family than boys and their counterparts from the western areas. Parents are generally concerned about the self-protection and puberty development for girls but neglected boys’ needs [29]. The reason for the regional difference might be that the eastern areas are more open to sexual topics than the western areas in China, especially the most developed and open city, Shanghai. Earlier in 2000, Shanghai was selected as a pilot city by the China Family Planning Association to conduct the China Youth Reproductive Health Project aimed at promoting the sexual and reproductive health of Chinese adolescents by offering school sexuality education [30].

Possibly due to inadequate sexuality education from their schools and family, adolescents turn to the Internet and peers to acquire sexual-related information. Some adolescents even use porn and other sexually explicit material as supplementary sources of sexual information [31,32]. In this study, the Internet and peers were the top two and top three sources of sexual-related knowledge. The Internet is full of unverified, low-quality, and fake sexual-related information. However, adolescents usually lack the ability to recognize the quality and accuracy of the information [33,34]. Therefore, they may be more likely to be misled by information obtained from the Internet, such as engaging in partner control, sexual assault, and risky sexual behaviors [35]. In this study, active porn watching was significantly associated with sexual intercourse (aOR = 2.63, 95% CI: 1.79–3.84), which is consistent with previous studies [32,36]. Of course, there may be an inverse association, where teens who engaged in sexuality might be more likely to watch pornography to satisfy their sexual impulses and “learning” needs. In our study, active porn watching was significantly associated with higher sexual-related knowledge (Table S2). However, evidence also shows that the presence of abuse, assault, and sexual exploitation in porn videos increases the risk of sexual coercion and harmful, gender-stereotypical attitudes [32,37,38]. Nowadays, pornographic culture has a tendency to transform into “pornographification of popular culture,” and soft pornography is mixed with entertainment products [39]. It is urgent to help adolescents improve their information literacy and decision-making skills [40]. Meanwhile, schools and families could prevent adverse outcomes of misleading media and pornography by delivering comprehensive SRH information to adolescents [41,42,43]. 

Although the proportion of sexual intercourse among VSSS in this study (5.8%) is lower than that of adolescents aged 15–17 in Taiwan (15.7%) and South Korea (6.76%) [44,45], it is higher than that of the national representative survey among the same-age adolescents in China’s urban areas (2.8%) [46]. Due to the sensitivity, however, most of the VSSS did not acquire information about sexual behavior, reproduction and contraception, and decision-making about sex from their schools and families, putting them at higher risk of unintended pregnancy, induced abortion, and STDs/HIV infection. Teachers and parents worry that talking about sex will encourage adolescents to engage in sexual intercourse. In this study, however, we did not observe association between school and family sexuality education and sexual intercourse. A large number of high-quality evidence also confirms that sexuality education in or out of schools does not increase sexual activity, sexual risk-taking behavior, or STI/HIV infection rates [47,48,49,50]. Adolescents’ sexual knowledge had a mild association with the experience of sexual intercourse in this study, possibly because they learned more sexual knowledge because of the experience. The finding may also suggest that adolescents are more likely to engage in safe sexual behaviors.

Obviously, the current “abstinence only” program cannot meet the needs of VSSS for sexuality education. Numerous studies suggested that comprehensive sexuality-education (CSE) programs based on gender, power, and human rights contribute to improved sexual knowledge and sexual self-efficacy, delayed initiation of sexual intercourse, reduced risk taking, and increased use of condoms and other contraceptives [8,49,50]. Considering the health risk of unsafe sexual intercourse among adolescents, school and family sexuality education with comprehensive coverage of sexual topics should be strengthened among VSSS to meet their needs for sexual related information, help them make healthy and responsible decisions, and allow them to refuse unsafe sexual behaviors.

### Strengths and Limitations

VSSS are a vulnerable but neglected sub-group of adolescents with regard to sexuality education in China. This study provides the latest evidence for promoting sexuality education among VSSS by exploring sexual-related knowledge and access to school and family sexuality education and its association with sexual intercourse among participants from the eastern and western areas of China. This study also had several limitations. First, this study was conducted among VSSS in the Shanghai and Shaanxi provinces. The findings might not be generalizable to other areas. Second, compared with offline surveys, online surveys are more likely to have duplicated records; however, we have made efforts to reduce such a situation by submitting screenshots for verification and reviewing background data. Third, as a cross-sectional study, this study could only suggest the association between sexual-related knowledge, school and family sexuality education, and sexual behaviors, rather than casual effects. Finally, response bias of self-reporting data cannot be avoided. However, efforts to ensure confidentiality were made to minimize the bias.

## Figures and Tables

**Table 1:** Socio-demographics of study participants by gender [$\bar{x}\pm s/\%$].

Characteristic	Total (*n* = 3180)	Boys (*n* = 1557)	Girls (*n* = 1623)	*p*
**Age in years**	16.9 ± 1.3	16.8 ± 1.3	17.0 ± 1.3	<0.001
**Region**				
Shaanxi	50.3	52.5	48.2	0.014
Shanghai	49.7	47.5	51.8	
**Local residents**				
Yes	80.6	84.3	77.2	<0.001
No	17.8	17.1	21.4	
Don’t Know	1.5	1.7	1.4	
**Living with parents**				
Both	76.6	73.5	80.0	<0.001
Only father	5.7	7.0	4.4	
Only mother	8.1	8.0	8.2	
None of them	9.7	11.5	7.9	
**Parents’ marital status**				
Married and living together	78.4	77.8	78.9	0.457
Others *	21.6	22.2	21.1	
**Family socio-economic status**				
Low	10.6	12.8	8.5	<0.001
Medium	63.4	61.0	65.7	
High	26.0	26.3	25.8	
Don’t Know	9.3	8.9	9.8	

*: including divorce, separation, and spouse death.

**Table 2:** Score on sexual-related knowledge by gender and region.

	Total	Sexual Physiology	STD/AIDS	Contraception	Sexual Abuse
**Gender**					
Boys	48.8 ± 22.3	43.6 ± 32.9	55.6 ± 24.5	42.7 ± 28.1	58.1 ± 44.3
Girls	53.8 ± 20.6	51.6 ± 30.5	60.0 ± 22.5	45.0 ± 25.5	74.4 ± 38.6
*p*	<0.001	<0.001	<0.001	0.018	<0.001
**Region**					
Shaanxi	47.6 ± 21.5	44.4 ± 31.4	55.1 ± 24.0	38.4 ± 26.1	64.4 ± 42.7
Shanghai	55.2 ± 21.1	51.0 ± 32.1	60.5 ± 22.9	49.4 ± 26.4	68.5 ± 41.8
*p*	<0.001	<0.001	<0.001	<0.001	0.005

**Table 3:** Topics covered in family and school sexuality education, by gender and region.

Variables	Gender			Region		
	Boys (*n* = 1557)	Girls (*n* = 1623)	*p*	Shaanxi (*n* = 1600)	Shanghai (*n* = 1580)	*p*
**Family sexuality education**						
Puberty development and health care	40.2	57.4	<0.001	49.0	48.9	0.966
Masturbation	6.7	1.8	<0.001	2.1	6.3	<0.001
Romantic relationships	33.1	46.3	<0.001	35.8	43.9	<0.001
Sexual dreams	5.7	3.3	0.002	3.3	5.6	0.002
The structure and function of human reproductive system	8.7	8.4	0.768	6.9	10.3	0.001
Sexual abuse and self-protection	9.5	27.4	<0.001	15.1	22.2	<0.001
Pregnancy/induced abortion/contraception	6.2	12.6	<0.001	6.3	12.6	<0.001
STD/AIDS	10.2	12.3	0.059	9.8	12.7	0.011
Gender identity/sexual orientation	13.4	20.1	<0.001	13.8	19.9	<0.001
Interpersonal relationships	55.2	61.2	0.001	63.2	53.3	<0.001
**School sexuality education**						
Puberty development and health care	81.6	85.4	0.004	80.1	87.1	<0.001
Values and decision making	66.6	58.4	<0.001	58.3	66.6	<0.001
Interpersonal relationships	65.2	67.4	0.186	62.4	70.3	<0.001
Sexuality and sexual behavior	44.9	41.8	0.082	34.2	52.6	<0.001
Reproduction and contraception	32.5	30.0	0.130	23.6	39.0	<0.001
STDs prevention	62.1	62.6	0.774	62.4	62.3	0.926
AIDS prevention	61.9	64.5	0.120	60.4	66.1	0.001
Saying no to drugs	77.4	78.3	0.532	76.2	79.6	0.022
Planning for the future	57.3	58.9	0.357	57.1	59.1	0.256

**Table 4:** Factors associated with sexual intercourse (OR, 95% CI).

Variables	Comparisons	Unadjusted			Adjusted		
		OR	95% CI		OR	95% CI	
Number of topics communicated with parents	Continuous	1.07	0.99	1.16	1.06	0.98	1.15
Number of topics in school sexuality education	Continuous	1.00	0.95	1.06	0.95	0.90	1.01
Sexual-related knowledge score	Continuous	1.02	1.01	1.03	1.01	1.00	1.02
Active porn watching	Yes vs. No	4.88	3.50	6.82	2.63	1.79	3.84

Adjusted for age, gender, region, whether local residents, family socio-economic status, parents’ marital status, living with parents, peers’ romantic relationships, and attitude toward middle school students’ sexual behavior.

## Data Availability

Any data requirements can be obtained by contacting the corresponding author.

## Supplementary Materials

The following supporting information can be downloaded at: https://www.mdpi.com/article/10.3390/children9081206/s1, Table S1: Questions about sexual-related knowledge and score; Table S2. Linear regression coefficients assessing the association between SRH knowledge and sources (β, 95%CI).

## Abbreviations

ASRH	Adolescent sexual and reproductive health
VSSS	Vocational secondary school students
CSE	Comprehensive sexuality education
WHO	World Health Organization
STDs	Sexually transmitted diseases
HIV	Human immunodeficiency virus
AIDS	Acquired immune deficiency syndrome
