# Supplementary material for: Sexual-Related Knowledge, School and Family Sexuality Education and Its Association with Experience of Sexual Intercourse among Vocational Secondary School Students in China
Source: Children (Basel). 2022 Aug 11;9(8):1206. doi: 10.3390/children9081206 (PMC9406409; doi:10.3390/children9081206)
Supplement: Supplementary file 1 [file children-09-01206-s001.zip › children-1819106-supplementary.pdf]

Table S1. Questions about sexual-related knowledge and score

	Score
**Physiological health**	**5**
Menstruation is regular, periodic bleeding from the shedding of the lining of the uterus	1
Nocturnal emission is a phenomenon in which semen is excreted after reaching a certain amount	1
Girls can get pregnant after menstruation	1
Boy with nocturnal emission may get pregnant from first sex	1
The uterus is where the fetus grows and develops	1
**STD/AIDS**	**11**
Heard of AIDS	1
Boys and girls may contracting HIV after first sex	1
Mosquito bites can transmit HIV/AIDS (false)	1
Condoms protect against pregnancy and STD/AIDS	1
A healthy looking person can't be HIV positive (false)	1
HIV testing facility	
Center for disease control and prevention	1
Public hospital	1
AIDS-prevention-related social organization	1
Heard of STDs	1
Sharing clothes, bathing utensils, toilets, etc. may transmit STD	1
Hepatitis B is a sexually transmitted disease	1
**Conception**	**11**
Know where to get contraceptives	1
Know following contraception methods	
Safe period	1
Contraception tablet/Pill	1
Condom	1
Vaginal sponge/Contraception cream/ Diaphragm	1
Implant	1
Sterilization	1
Emergency contraception	1
Intrauterine device	1
Injection	1
Others	1
**Sexual abuse**	**2**
Only physical touching can be considered sexual harassment, not include uncomfortable sexual remarks, graphic displays, eyes expression, or gestures (false)	1
It is not a compulsive sexual behavior if not forced by physical violence (wrong)	1

Table S2. Linear regression coefficients assessing the association between SRH knowledge and sources (β, 95%CI)

Variables	Comparisons	Unadjusted			Adjusted		
		β	95%CI		β	95%CI	
Active porn watching	Yes vs. No	11.84	9.38	14.30	10.00	7.70	12.30
Sources: Parents	Yes vs. No	5.45	3.84	7.06	-0.70	-2.33	0.94
Sources: Schools/teachers	Yes vs. No	7.89	6.36	9.41	6.24	4.77	7.73
Sources: Siblings/relatives	Yes vs. No	9.09	7.16	11.02	-0.98	-3.01	1.06
Sources: Peers	Yes vs. No	10.66	9.20	12.12	6.82	5.36	8.29
Sources: Books/magazines/newspapers	Yes vs. No	12.68	11.18	14.18	6.85	5.22	8.48
Sources: TV/radio	Yes vs. No	9.88	8.33	11.45	0.76	-0.94	2.46
Sources: SRH professionals	Yes vs. No	8.31	6.57	10.05	3.15	1.36	4.93
Sources: Internet	Yes vs. No	10.55	9.09	12.02	6.29	4.84	7.74
Sources: Community sourced materials	Yes vs. No	8.71	6.91	10.50	2.43	0.60	4.28

Adjusted for age, gender, region, whether local residents, family socio-economic status, parents' marital status, living with parents
